# Palmar skin conductance variability and the relation to stimulation, pain and the motor activity assessment scale in intensive care unit patients

**DOI:** 10.1186/cc12571

**Published:** 2013-03-19

**Authors:** Anders C Günther, Matteo Bottai, Anna R Schandl, Hanne Storm, Patrik Rossi, Peter V Sackey

**Affiliations:** 1 Section of Anesthesiology and Intensive Care Medicine, Department of Physiology and Pharmacology, Karolinska Institutet, 17177 Stockholm, Sweden; 2 Unit of Biostatistics, Department of Environmental Medicine, Karolinska Institutet, 17177 Stockholm, Sweden; 3 Section for Simulation, Faculty of Medicine, Division Rikshospitalet, University of Oslo, 0027 Oslo, Norway

## Abstract

**Introduction:**

Many intensive care unit (ICU) patients describe pain and other adverse feelings that may impact long-term psychological morbidity. Sympathetically mediated palmar skin conductance variability is related to emotionally induced perspiration and correlates with pain levels in the perioperative setting but has not been studied in ICU patients.

**Methods:**

Twenty non-intubated and 20 intubated general ICU patients were included in this observational study. Patients were monitored with the MED-STORM Pain Monitoring System^®^. The number of skin conductance fluctuations per second (NSCF) was measured in parallel with bedside observation during one hour of intensive care, including rest, procedures and patient-staff interactions. Arousal-agitation level was monitored with the motor activity assessment scale (MAAS). Pain was monitored with the numeric rating scale (0 to 10) in patients able to communicate or by observation in patients unable to communicate.

**Results:**

In non-intubated patients, NSCF increased with increasing stimulation/pain but also with higher MAAS (*P *= 0.002). An interaction effect was found, with increased NSCF response to stimulation/pain with increasing MAAS (*P *< 0.001).

In intubated patients, NSCF increased significantly with increasing stimulation/pain (*P *< 0.001). In contrast to non-intubated patients, no difference in NSCF between MAAS levels was found for any given degree of stimulation in intubated patients.

**Conclusions:**

In critically ill patients, NSCF may be more useful evaluating emotional distress rather than pain alone. It needs to be assessed whether NSCF monitoring is clinically useful and whether controlling emotional distress with the aid of such monitoring may impact on patient care and outcomes.

## Introduction

Clinical practice guidelines for sedation and analgesia have been developed in order to achieve and maintain comfort and safety in critically ill patients [1]. Still, many intensive care unit (ICU) patients remember having anxiety and unrelieved pain when interviewed after the ICU stay [2,3]. Such negative memories have been associated with adverse long-term outcomes [4].

Emotional stress is associated with increased activity in the sympathetic nervous system. This increased activity includes the activation of palmar sweat glands [5]. Sweat gland activation in response to emotional stress is believed to originate in parts of the limbic system [5]. Via brainstem activation, cholinergic transmission in paravertebral ganglia increases and eventually leads to activation of muscarinic sweat gland receptors [5]. Increased palmar moisture leads to altered electrical conductance of the palm [5-7]. Basal skin moisture level, skin quality and surrounding temperature vary in different individuals and settings, making absolute palmar skin conductance highly individual [7]. In contrast, skin conductance variability - mediated by intermittent palmar sweat release and reuptake - more directly mirrors ongoing sympathetic nerve activity to palmar sweat glands, as measured with neurography [5]. Peripherally administered vasopressors and inotropes, such as epinephrine, norepinephrine or dobutamine, do not pass the blood-brain barrier and have little effect on muscarinic receptors. For these reasons they are likely to have little impact on palmar sweat gland activation.

In order to measure ongoing palmar sweat gland activation in response to emotional stress, a measure of skin conductance variability (number of skin conductance fluctuations, NSCF) has recently been developed and evaluated [7,8]. In a study of intraoperative stimulation, NSCF changed faster and with greater sensitivity during stimulation than did more classical indicators of intraoperative pain, such as blood pressure or heart rate [9]. NSCF has also been found to correlate with postoperative patient ratings of pain [10,11].

To our knowledge, skin conductance variability has not been evaluated as a measure of pain in critically ill adults. We performed this observational study in order to explore the relationship between skin conductance variability (referred to as NSCF) and varying degrees of stimulation, pain and level of arousal-agitation in a mixed ICU patient population.

## Materials and methods

### Design, inclusion and exclusion criteria

This prospective observational study was performed at the general ICU, a mixed medical and surgical ICU at Karolinska University Hospital Solna, Stockholm. The study was approved by the local Ethical Review Board in Stockholm (Regionala Etikprövningsnämnden I Stockholm). Forty critically ill patients age above 17 years, were included (20 non-intubated and 20 intubated). Patients were excluded if they were diagnosed with neuro- or myopathy, were receiving neuromuscular blocking agents, or were treated with atropine or glycopyrrolate the same day, as such treatments or conditions could potentially make clinical assessment difficult or affect muscarinic sweat gland receptor activity. Written informed consent from the participants or their closest relatives was obtained.

### Skin conductance variability monitoring

Skin conductance variability was monitored during one hour of routine daytime intensive care nursing and treatment. A skin conductance monitor, Med-Storm Pain Monitoring System^® ^(MED-STORM Innovation AS, Oslo, Norway) was used for this purpose. Three single use Ag/AgCl electrodes (MED-STORM Innovation AS, Oslo, Norway) were attached to the palmar surface of the patient's hand: on the thenar eminence (current electrode), hypothenar eminence (measurement electrode) and just below the second and third digits (reference electrode). The hand least likely to be moved was chosen to minimize movement artifacts. Skin conductance variability was measured by alternating current at 66 Hz and an applied voltage of 50 mV.

Skin conductance variability was measured as the number of skin conductance fluctuations per second (NSCF). The cutoff for identifying skin conductance fluctuations was set with skin conductance troughs and peaks of an amplitude > 0.02 microsiemens (μS), as in previous studies of NSCF in the perioperative setting [9-11]. The measurement window - the sampling time from which the mean NSCF value was calculated - was 15 seconds. The refreshing time, the time a new measurement window was analyzed, was one second. Incoming data were displayed on-line on a laptop connected to the monitor via a standard serial port and stored for subsequent analysis. Monitoring was not blinded so that the observers could see the skin conductance curve and the values of NSCF and note when artifacts disturbed registration.

### Clinical assessment

Parallel with NSCF monitoring, the level of stimulation/pain and arousal-agitation were assessed.

#### Stimulation/pain assessment

For awake and communicative patients, pain level on the 11-point numeric rating scale (NRS 0 to 10) [12] was asked for and noted during procedures. For patients unable to communicate, the patient's behavior at rest and during procedures was observed.

Observations of stimulation/pain were categorized in four groups:

A. No stimulation. The patient was lying undisturbed, without any observed or reported pain (see D).

B. Mild stimulation without observed or reported pain. The patient was being spoken to or procedures, such as gentle washing, were performed or the patient made slight movements without observed or reported pain.

C. Potentially painful stimulation without observed or reported pain. The patient did not report or show signs of pain but was exposed to any of the following procedures or conditions:

1. Needle stick.

2. Turning of the patient.

3. Suction of the mouth, hypopharynx or endotracheal tube.

4. Unsynchronized with the ventilator or abnormal breathing pattern.

5. Dressing of wound.

D. High pain rating or overt expression of pain in rest or during stimulation/procedure. The patient expressed pain verbally (NRS above 3). If the patient could not rate pain with the NRS the following signs were considered indicative of pain or discomfort:

1. Facial grimacing.

2. Moaning or groaning.

3. Localizing painful area, withdrawing from touch or resisting potentially painful movement or procedure.

#### Arousal/agitation assessment

Patients' arousal/agitation level was assessed with the Motor Activity Assessment Scale (MAAS) scale (Table 1) [13], the scale used in our general ICU.

**Table 1:** Motor Activity Assessment Scale (MAAS) [14]

Score	Description	Definition
**0**	Unresponsive	Does not move with noxious stimuli
**1**	Responsive only to noxious stimuli	Open eyes, raises eyebrows or turns head toward stimulus; moves limbs with noxious stimulus
**2**	Responsive to touch or name	Open eyes, raises eyebrows or turns head toward stimulus when touched or name is loudly spoken
**3**	Calm and cooperative	No external stimulus in required to elicit movement; adjusts sheets or clothes purposefully, follows commands
**4**	Restless and cooperative	No external stimulus in required to elicit movement; picks at sheets or tubes, uncovers self, follows commands
**5**	Agitated	No external stimulus in required to elicit movement, attempts to sit up or moves limbs out of bed, does not consistently follow commands (for example, will lie down when asked to but soon reverts back to attempts to sit up or move limbs out of bed)
**6**	Dangerously agitated, uncooperative	No external stimulus in required to elicit movement; pulls at tubes or catheters, thrashes side to side, strikes at staff, tries to climb out of bed, does not calm down when asked.

### Data collection

During the hour of monitoring, typical procedures were washing, turning the patient, physiotherapy and in some cases also invasive procedures, such as intravenous line insertion or bronchoscopy. At the time of the event or procedure, an on-line mark on the laptop monitor was made and the NRS or observed stimulation/pain level and MAAS level was noted. Comments describing the event or procedure were made for each observation. The mean NSCF value, representing the 15-second window at the time of the event, was later extracted for analysis. When the skin conductance signal quality was below 80% (as a result of distortion of the reference signal or a change of the integrity of the measured signal) or connection was temporarily lost, the event was registered as "artifact", as were situations when the patient moved the NSCF measurement hand.

### Statistical analysis

Unlike the non-intubated patients, the intubated patients could not speak and more often received sedative and opiate infusions. The intubated and non-intubated patients were, therefore, analyzed separately. Random-effects regression models were used to analyze NSCF over different pain and MAAS levels. In the regression models, stimulation and MAAS were introduced as numeric variables. Statistical significance level was set at *P *< 0.01. Analyses were performed with Stata version 12 (StataCorp, College Station, TX, USA).

## Results

Patient demographics are presented in Table 2. In total, the non-intubated patients contributed 715 registrations and the intubated patients 735 registrations. Room temperatures during the study ranged between 22° and 24°C. Patient temperatures ranged between 37.4° and 40.0°C. Sedative and analgesic drugs were more frequently used in intubated than in non-intubated patients (Table 3). The MAAS score ranged between 2 and 5 for non-intubated patients and between 0 and 4 in the intubated patients. Fourteen of the 20 non-intubated patients rated their pain with the NRS.

**Table 2:** Patient demographics

	Intubated	Non-intubated
**Age; mean (SD)**	60 (16)	55(18)
**sex;**		
**Female**	2	7
**Male**	18	13
ICU day*; median (range)	3 (1 to 13)	2 (1 to 19)
**Diagnosis group (n);**		
**Medical**	4	6
**Surgical**	4	4
**Trauma**	4	6
**Sepsis**	8	4

*Day of registration.

**Table 3:** Sedation and/or analgesia at the time of NSCF registration

Sedation/analgesia		Intubated *n *= 20	Non-Intubated *n *= 20
**Opiates**	**infusion**	10	0
	**intermittent**	6	5
**Bensodiazepines**	**infusion**	9	0
	**intermittent**	1	0
**Propofol**	**infusion**	6	0
	**intermittent**	4	0
**Clonidine**		6	3
**Epidural**		2	5

**Figure 1:**
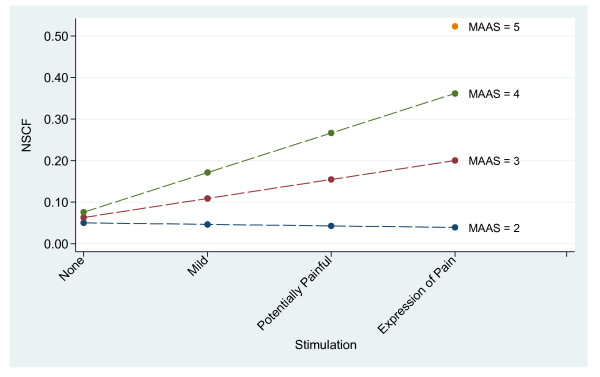
**Skin conductance variability (NSCF) in relation to stimulation and Motor Activity Assessment Scale, non-intubated patients**.

In non-intubated patients, higher levels of stimulation/pain were associated with higher NSCF (*P *= 0.002) for all MAAS levels except MAAS 2 (Figure 1, Table 4). There was significant interaction between stimulation and MAAS level (*P *< 0.001), with an increased NSCF response to stimulation with increasing MAAS (Figure 1, Table 5).

**Table 4:** NSCF in relation to MAAS and degree of stimulation in non-intubated patients

	MAAS 2 *n *= 30	MAAS 3 *n *= 589	MAAS 4 *n *= 92	MAAS 5 *n *= 4
**No stimulation** ***n *= 77**	0,01 *n *= 14	0.07 *n *= 98	0,12 *n *= 5	No observation
**Mild stimulation** ***n *= 165**	0.08 *n *= 10	0.10 *n *= 256	0.18 *n *= 73	No observation
**Potentially painful stimulation** ***n *= 53**	0.30 *n *= 2	0.21 *n *= 66	0.46 *n *= 7	No observation
**Expression of pain** ***n *= 112**	0.03 *n *= 4	0.21 *n *= 169	0.50 *n *= 7	0.48 *n *= 4

Cross-tabulated mean (above) and count (below).

**Table 5:** NSCF in relation to MAAS and degree of stimulation in intubated patients

	MAAS 0 *n *= 11	MAAS 1 *n *= 339	MAAS 2 *n *= 221	MAAS 3 *n *= 96	MAAS 4 *n *= 68
**No stimulation** ***n *= 126**	0.04 *n *= 7	0.05 *n *= 85	0.08 *n *= 16	0.28 *n *= 18	No observation
**Mild stimulation** ***n *= 329**	0.09 *n *= 3	0.07 *n *= 110	0.06 *n *= 116	0.18 *n *= 64	0.24 *n *= 36
**Potentially painful stimulation** ***n *= 233**	0.07 *n *= 1	0.14 *n *= 129	0.10 *n *= 68	0.29 *n *= 12	0.26 *n *= 23
**Expression of pain** ***n *= 47**	No observation	0.14 *n *= 15	0.10 *n *= 21	0.23 *n *= 2	0.32 *n *= 9

Cross-tabulated mean (above) and count (below).

**Figure 2:**
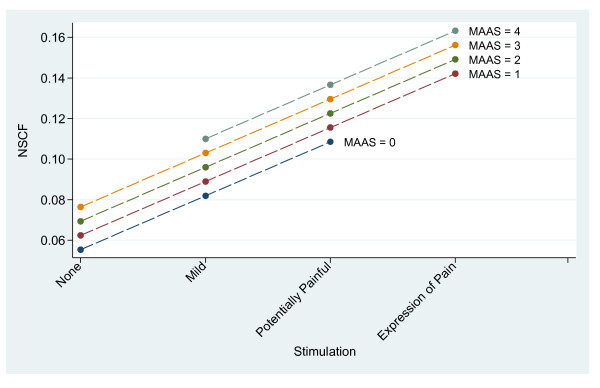
**Skin conductance variability (NSCF), in relation to stimulation and Motor Activity Assessment Scale, intubated patients**.

For intubated patients, increasing stimulation/pain was associated with increased NSCF (*P *< 0.001) at all MAAS levels (Figure 2, Table 3). In contrast to non-intubated patients, increasing MAAS level was not significantly associated with an increase in NSCF (*P *= 0.64) (Figure 2).

Artifacts precluded adequate registration 83 times (11.3% of observations) in the intubated patients and 206 times (28.8% of observations) in the non-intubated patients.

## Discussion

To our knowledge, this is the first study evaluating skin conductance variability in critically ill adult patients. The main findings were that NSCF generally increased with the degree of stimulation/pain, an observation common for both patient groups.

The highest NSCF readings were found in non-intubated patients with high MAAS level and with overt expression of pain. A higher degree of arousal or agitation was associated with a stronger NCSF response to stimulation/pain (Figure 1). This finding can be interpreted as similar to that of higher subjective pain rating during states of elevated stress or anxiety [14]. Previous studies of NSCF in the anesthesia or postoperative setting have focused on detecting pain [9-11]. While postoperative patients might experience pain as an isolated negative perception, it is likely that ICU patients that are awake (that is, MAAS > 2) are aware of their severity of illness and being in the ICU. Such awareness may potentially contribute to emotional stress [15] and subsequent NSCF response to stimulation/pain.

In the intubated patients, NSCF values were lower overall, but still with increasing NSCF with increasing stimulation. MAAS level change did not significantly alter the NSCF response to stimulation or pain (*P *= 0.64). One difference between study groups, besides endotracheal intubation, was that the intubated patients received more sedatives and analgesics (Table 3). As a desired pharmacological effect, these drugs sedate the patient, potentially affecting awareness of the critical situation. Our interpretation of the differences between the two groups is that ICU patients that are awake and increasingly aware, aroused or agitated are more reactive to pain than those calm or less rousable.

We find it of some interest that intubated patients with MAAS 3 and without any on-going activity or stimulation had a mean NSCF of 0.28 (Table 5), a level associated with high pain rating in a previous study of NSCF and postoperative pain [11]. In that study, NSCF above 0.2 was associated with an NRS pain rating of 4 and above [11]. It may be that these intubated and awake patients were in greater discomfort or pain than what was apparent from the clinical observation. However, only four patients contributed to these data, MAAS 3 without ongoing activity/stimulation (of which one patient contributed with 13 of 18 observations), which precludes firm conclusion of this finding.

The number of registrations when artifacts were present was relatively high (28.8% in non-intubated patients and 11.3% in intubated patients). In some patients the electrodes fell off due to movement and stretching of the wires, or due to excessive sweating. Electrode adhesion problems and movement artifacts may possibly be reduced with better electrodes and wrapping of the hand [16].

Generally, we interpret our study findings as that NSCF appears to mirror distress rather than pain alone, which is a limitation from a pain monitoring perspective. Instead, skin conductance variability monitoring might be a means of monitoring discomfort or distress in ICU patients. The level of comfort and sedation, as well as sedative choice may affect memories from the ICU [17,18], and thereby potentially impact psychological well-being and long-term quality of life [18,19]. Whether distress indicated with skin conductance variability monitoring is deleterious for patients' perception of and memories from the ICU stay and subsequent psychological outcome deserves further study, with longer-term skin conductance monitoring and possibly including pharmacological and non-pharmacological interventions.

### Limitations

A major limitation in our study was our classification of stimulation/pain in non-communicating patients. At the outset of the study, we did not have a validated Swedish pain monitoring instrument available. We used a non-standardized way of assessing pain in these patients, namely by observing facial grimacing, body movements or sounds as indicators of pain in patients unable to communicate verbally, which may have led to some degree of misclassification. Recently, pain monitoring instruments for poorly communicating patients have been developed and validated [20,21], with the Critical Care Pain Observational Tool now also available in Swedish [22].

Another limitation is that the study observers were not blinded for online NSCF levels. The monitor was new for the observers and we felt that information displayed was important in this pilot study. Online information included electrode status and signal quality, the time marker and online "notepad". For a more formal validation study, including specificity and sensitivity analyses, a validated pain assessment tool or a composite pain and agitation score would be appropriate to use, as well as blinding of skin conductance levels.

Finally, while NSCF has been shown to decrease with analgesic treatment of pain in postoperative patients [11], our protocol did not include evaluation of changes in NSCF in response to sedation or analgesia due to emotional stress or pain. Such evaluation would have been valuable and is warranted in future studies.

## Conclusions

Palmar skin conductance variability increases with increasing stimulation in critically ill patients. In non-intubated patients increasing MAAS levels are associated with increased NSCF responses to stimulation/pain. This interaction is not seen during mechanical ventilation and sedation. Future studies of skin conductance variability are needed, to investigate if skin conductance variability monitoring can aid ICU clinicians in the general management of emotional stress due to pain and other adverse feelings, and if such monitoring and subsequent interventions are associated with improved patient outcomes.

## Key messages

• Skin conductance variability as measured as NSCF, increases with increasing stimulation/pain in critically ill ICU patients.

• In less sedated and non-intubated ICU patients, increasing arousal and agitation increases the NSCF response to stimulation.

• Further research is warranted to investigate potential clinical benefits of NSCF monitoring in ICU patients.

## Abbreviations

μS: microsiemens; ICU: Intensive Care Unit; MAAS: Motor Activity Assessment Scale; NRS: numeric rating scale; NSCF: number of skin conductance fluctuations

## Competing interests

Hanne Storm has a potential competing interest as a part owner, CEO and share-holder in Med-Storm Innovation AS, and has contributed to the development of the skin conductance monitor used in the study. Med-Storm Innovation AS has patents on the skin conductance equipment used in this study for measuring pain and sedation level. The other authors report no competing interests.

## Authors' contributions

AG, PS, AS, HS and PR contributed to the conception and design of the study. AG, AS and PS collected data. AG, PS, PR, MB and HS participated in data analysis and interpretation. AG and PS drafted the manuscript and all authors were involved in the revision and final approval of the submitted manuscript.
